# Epithelial-myoepithelial carcinoma: Retrospective analysis of the case series from a reference center in São Paulo, Brazil^☆^^☆☆^

**DOI:** 10.1016/j.bjorl.2025.101615

**Published:** 2025-05-29

**Authors:** Gabriela Miglioranza Gonçalves Luccas, Kim Soares Marinho, Lara Caroline Cardoso, Maria Paula Spegiorin de Oliveira, Renato Nobutaka Gotoda, Dorival de Carlucci Junior, Luiz Paulo Kowalski

**Affiliations:** a Universidade de São Paulo (USP), Faculdade de Medicina (FM), Hospital das Clínicas (HC), São Paulo, SP, Brazil; b Universidade Anhembi-Morumbi, Faculdade de Medicina, São Paulo, SP, Brazil

**Keywords:** Salivary glands, Parotid gland, Malignant parotid neoplasms, Carcinoma

## Abstract

**Objective:**

Given the recent increase in diagnoses in our service, a referral center in São Paulo, this study aims to analyze the case series at this center and compare it with the existing literature. The objective is to comprehend the characteristics and prognosis of this disease and provide insights to enhance its treatment.

**Methods:**

Confirmed cases of EMC were selected at the Hospital das Clínicas da FMUSP between 2011 and 2021. Fourteen cases were screened and subjected to retrospective analysis of medical records for data collection.

**Results:**

The analysis covered patient and disease characteristics. Of the total, 64.3% were female, with an average age of 71.33 years. The parotid gland was the predominant primary site (92.8%). Concerning staging, there was a significant prevalence in stages III and IVa. Among the cases, 12 underwent surgery, and eight patients received adjuvant therapy.

**Conclusion:**

Despite EMC being a low-grade neoplasm, our case series, while aligning with much of the existing literature, revealed a more advanced presentation. This raises questions about whether this aggressiveness is due to late diagnosis or whether the neoplasm is less indolent than presumed.

**Level of evidence:**

Level IV.

## Highlights

• The tumor was more frequent in female, with an average age of 71.33 years.
• The parotid gland was the predominant primary site (92.8%).
• There was a significant prevalence in stages III and IVa.
• Most received adjuvant treatment after surgery as the mainstay treatment option.

## Introduction

Epithelial-Myoepithelial Carcinoma (EMC) is a rare and biphasic malignant neoplasm of the salivary glands, particularly affecting the parotid gland.^1^

The standard treatment remains uncertain because of its low incidence in the population, representing approximately 1% of salivary gland neoplasms.^2^ EMC was first described by Donath et al.^3^ in 1972, and it was incorporated into the World Health Organization (WHO) classifications only in 1991.^4^ Because of the rarity of this pathology, most information about it is based on case reports, totaling 822 cases described in the literature until 2018.

Despite being a highly uncommon disease, in recent years, we have observed a significant increase in the frequency of diagnoses in our service. Therefore, through this study, we seek to analyze the case series of a reference center in São Paulo and compare it to the existing literature. Our goal is not only to understand the disease’s characteristics and prognosis more comprehensively but also to provide more information to continually improve the treatment given to patients.

**Fig. 1:**
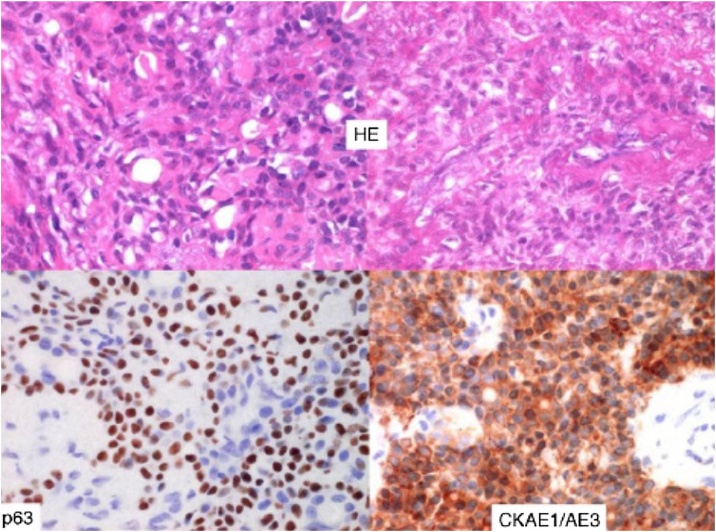
Image highlighting the characteristic structure of Epithelial-Myoepithelial Carcinoma. On the bottom layer, the myoepithelial and epithelial components are emphasized (from left to right).^6^

Histologically, EMC is characterized by a biphasic organization, consisting of ductal epithelial cells surrounded by clear myoepithelial cells, a distinctive feature of the disease,^4^, ^5^ as illustrated in Fig. 1. Regarding Immunohistochemistry (IHC), some studies have reported the presence of cytokeratins AE1/AE3 and CAM 5.2 – related to the intraductal component, and p63, Vimentin, and Smooth Muscle Actin – related to the myoepithelial component.^6^ Both macroscopically and histologically, the neoplasm presents as encapsulated, with minimal extravasation described.

The literature describes its incidence as being slightly more common in female patients aged >60-years, without well-defined risk factors.^7^, ^8^

Clinically, it usually presents as a painless mass with progressive growth, seldom showing cutaneous or neural involvement. The parotid gland is the most frequently affected site, followed by the submandibular gland. It is crucial to emphasize that, despite the higher incidence in the parotid gland, EMC is not exclusive to this gland and has been described in other major salivary glands, minor salivary glands, nasal cavity and paranasal sinuses, oropharynx, and larynx.^9^, ^10^

Similar to other salivary gland neoplasms, Ultrasonography (US) is often the first and most accessible examination, assisting in delineating the boundaries of the lesion, especially in superficial lobe lesions, evaluating lymph nodes, and differentiating between cystic and solid lesions. However, the US assessment is not validated for differentiating between histological types. It is commonly combined with Fine-Needle Aspiration (FNA), which significantly aids in determining the malignancy of the sample, with a sensitivity of 90.9% and a specificity of 87.1%. However, it still presents limitations in identifying the specific histological type of the lesion.^11^

When discussing the standard treatment for this pathology, as with most salivary gland lesions, the surgical approach is considered the basis of treatment. The proposal of lesion resection with margins is well described, but there is little consensus on the need for combined elective cervical dissection, with suggestions of performing it in patients with locally advanced Tumors (T3/T4) or with facial nerve involvement.^12^ Regarding adjuvant therapy, there is a lack of randomized studies on the benefits of using radiotherapy, but the literature reports good results of its use for locoregional control of the disease.^13^

As for prognosis, the specific disease survival rates at 5- and 10-years are 91.3% and 90.2%, respectively, considering the salivary glands as the primary site.^14^ However, considering other sites, which are less prevalent and have lower survival rates, the figures are 72.7% and 59.5% at 5- and 10-years, respectively. Moreover, it is worth noting that statistically significant factors associated with survival were age, race, staging by the American Joint Committee on Cancer (AJCC) – T, N, and M, and treatment modality.^2^

## Materials and methods

For this study, cases with an anatomopathological confirmation of EMC were selected from the Hospital das Clínicas Complex of the School of Medicine of the University of São Paulo (HC-FMUSP) ‒ a reference service located in São Paulo, state of São Paulo, Brazil ‒ between 2011 and 2021. This included the Central Institute of HC-FMUSP (ICHC) and the Cancer Institute of the State of São Paulo (ICESP).

After analyzing the pathological anatomy results from these institutions, 14 cases were selected, with eight coming from ICHC and six from ICESP. These were subjected to a retrospective analysis of medical records to obtain the data presented below.

## Results

We analyzed the data in two aspects: patient characteristics and disease characteristics. Regarding the former, among the selected cases, 64.3% were female and 35.7% were male, resulting in a ratio of 1.8:1 (F:M).

Concerning race, there was a higher prevalence in white patients, accounting for 42.5% of cases, followed by other races (brown, black, yellow, and not informed), each representing 14.2%. The average age among the cases was 71.33-years, with a median of 61, ranging from 36- to 93-years.

As for the disease characteristics, there was a significant prevalence of the parotid gland as the primary site, observed in 92.8% of cases, compared with only 7.1% in the oropharynx, with no occurrence in other sites. Finally, regarding staging, the results were 7.1%, 14.2%, 28.5%, 21.4%, and 7.1% for stages I, II, III, IVa, and IVc, respectively.

Only one patient was referred to non-surgical treatment by patient choice. This case involved a tumor with an epicenter in the oropharynx, cT4aN1M0, initially treated with concurrent chemotherapy and radiotherapy in 2021. However, the patient progressed with pulmonary disease in 2022 and was then referred to palliative chemotherapy.

For the surgical cases, the procedures included eight partial parotidectomies, one partial parotidectomy with elective cervical dissection levels I to III, two total parotidectomies with elective cervical dissection and the necessity for facial nerve reconstruction by plastic surgery, and one total parotidectomy extended to the skin and the mastoid with elective cervical dissection.

Regarding adjuvant therapy, of the 14 patients, eight were recommended additional treatment based on the presence of perineural invasion, angiolymphatic invasion, and advanced staging (T3/T4). Of these eight patients, one discontinued treatment because of toxicity, and one was lost to follow-up after the recommendation, eventually experiencing local recurrence and pulmonary disease progression, requiring a new local surgical approach followed by radiotherapy, as well as pulmonary metastasectomy by thoracic surgery.

In terms of patient outcomes, one patient was lost to follow-up after undergoing FNA and was not treated at our service, one was lost to follow-up during the COVID-19 pandemic, three have already been discharged from an oncological perspective, and one developed a pulmonary metastasis in 2019, undergoing metastasectomy in 2020.

## Discussion

Despite the increasing number of cases described, little consensus has been reached about EMC, and here we hope to contribute more data to the existing literature. In our case series, we found many similarities with the literature described to date, which we will discuss below.

**Fig. 2:**
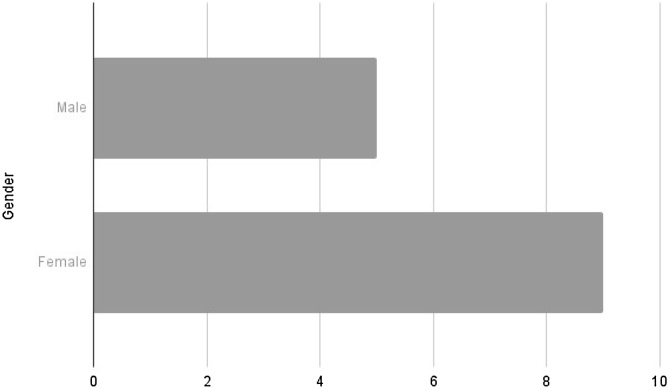
Gender distribution.

Both Vázquez et al.^14^ and Gore^2^ have described a higher prevalence of EMC in females ‒ a ratio also present in our case series ‒ at a ratio of 1.8:1 (F:M), as shown in Fig. 2.

**Fig. 3:**
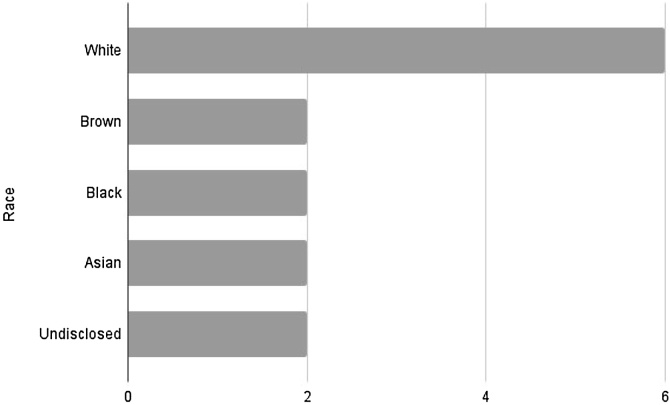
Racial distribution.

**Fig. 4:**
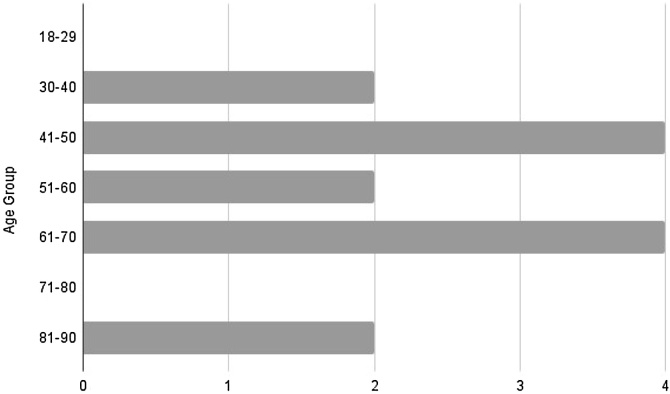
Age distribution.

Concerning race, our sample, as the general literature, showed a higher prevalence among self-declared white individuals. However, despite the second most affected race being black in general, our case series showed equal distribution among other races, as shown in Fig. 3. Regarding age distribution, we observed an average patient age of 71.33-years, in line with previously described data, with a large number of cases around the 7th decade of life (Fig. 4).

**Fig. 5:**
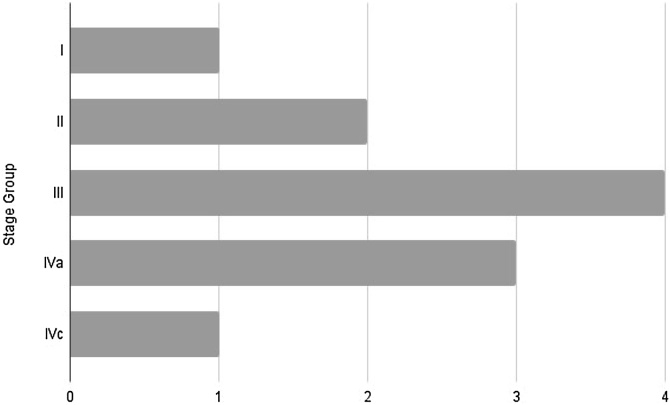
Distribution of primary sites.

Among the 14 cases analyzed, the most common primary site was the parotid gland, followed by the oropharynx, as per Fig. 5. Although we align with the literature in terms of the higher prevalence of cases in the parotid gland, we observed a discrepancy in our data; unlike what is demonstrated in the literature, we did not have cases in other salivary glands – according to the current literature, the submandibular gland would be the second most common site. However, because of the limited number of cases, we cannot make statistically significant assertions about this distribution. Moreover, it is important to stress that Gore,^2^ in updating the case series initially studied by Vázquez et al.,^14^ also included cases from primary sites distant from the head and neck region, which was not considered in our work.

**Fig. 6:**
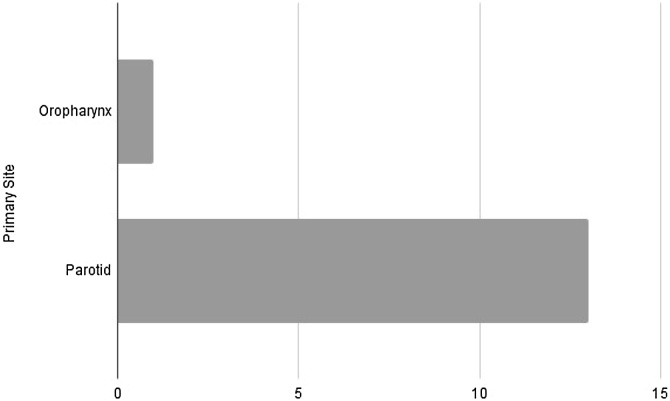
Staging distribution.

Finally, regarding staging, we obtained a result that differs from what is described in the literature, as shown in Fig. 6. According to the reviewed literature, EMC is defined as an indolent, low-grade, malignant neoplasm, with stage I being the most prevalent at anatomopathological diagnosis.

However, in our case series, we observed a prevalence of patients with stage III, followed by stage IVa and, in third place, stage II. As previously described, there was a significant number of patients requiring adjuvant therapy (57%) and of metastatic cases (28%) in our sample, indicating more advanced cases than we expected. Given these findings, we observe that our sample presented more severe cases at diagnosis, raising the question: Is this due solely to late diagnosis, or can we suspect that EMC is not as indolent as previously thought?

## Conclusion

Epithelial-Myoepithelial Carcinoma (EMC) is a rare, low-grade, malignant neoplasm of the salivary glands in which the diagnosis is most often established only after the anatomopathological analysis of the surgical specimen, often requiring supplementation with IHC because of its similarities to other salivary gland pathologies.

Even in a service that is a reference in Latin America, the sample size is not large enough to draw more robust conclusions about EMC. The analysis of our case series not only allowed a more comprehensive understanding of the cases encountered in a quaternary service connected to the public health system but also corroborated some findings previously described in the literature. However, faced with a more aggressive behavior than what had been characteristic of the disease so far, we hope to stimulate further studies on EMC to provide our patients with possibilities for more accurate diagnosis and increasingly targeted and specific treatment for this pathology.

## Declaration of competing interest

The authors declare no conflicts of interest.
